# Advances and challenges in conventional and modern techniques for halal food authentication: A review

**DOI:** 10.1002/fsn3.3870

**Published:** 2023-12-07

**Authors:** Ifrah Usman, Saima Sana, Muhammad Afzaal, Ali Imran, Farhan Saeed, Aftab Ahmed, Yasir Abbas Shah, Muniba Munir, Huda Ateeq, Atka Afzal, Iqra Azam, Afaf Ejaz, Gulzar Ahmad Nayik, Mahbubar Rahman Khan

**Affiliations:** ^1^ Department of Food Sciences Government College University Faisalabad Faisalabad Pakistan; ^2^ University Institute of Food Science and Technology, The University of Lahore Lahore Pakistan; ^3^ Department of Nutritional Sciences Government College University Faisalabad Faisalabad Pakistan; ^4^ National Institute for Biotechnology & Genetic Engineering Faisalabad Faisalabad Pakistan; ^5^ Department of Food Sciences Government College Women University Faisalabad Faisalabad Pakistan; ^6^ Department of Food Science and Technology Government Degree College Shopian Shopian Jammu and Kashmir India; ^7^ Department of Food Processing and Preservation Hajee Mohammad Danesh Science & Technology University Bangladesh

**Keywords:** alcohol, conventional methods, halal food authentication, modern techniques, pork, religious ethics

## Abstract

Food is one of the most necessary needs since human civilization. For Muslims, it is mandatory to consume halal food. From a halal authentication perspective, adulteration of food products is an emerging challenge worldwide. The demand for halal food consumption has resulted in an ever‐increasing need for halal product validity. In the market, there are several food products in which actual ingredients and their source are not mentioned on the label and cannot be observed by the naked eye. Commonly nonhalal items include pig derivatives like lard, pork, and gelatin derivatives, dead meats, alcohol, blood, and prohibited animals. Purposely, various conventional and modern methods offer precise approaches to ensure the halalness and wholesomeness of food products. Conventional methods are physiochemical (dielectric) and electrophoresis. At the same time, modern techniques include high‐pressure liquid chromatography (HPLC), gas chromatography (GC), electronic nose (E‐Nose), polymerase chain reaction (PCR), enzyme‐linked immunosorbent assay (ELISA), differential scanning calorimetry (DSC), nuclear magnetic resonance (NMR), near‐infrared (NIR) spectroscopy, and Fourier transform infrared (FTIR) spectroscopy. This review intends to give an extensive and updated overview of conventional and modern analytical methods for ensuring food halal authenticity.

## Graphical abstract

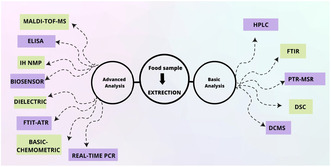

## 1 INTRODUCTION

According to Islamic teachings, the Arabic word “halal,” which appears in the holy Qur'an, can be translated into English as “permissible”. Every Muslim's faith is based on a basic understanding of what qualifies as halal. Although the Qur'an considers anything lawful or legal as “halal,” the phrase is most commonly used for acceptable food (Mortas et al., 2022). Foods that are forbidden or illegal, like alcohol and pork, are referred to as “haram”. Halal food is properly labeled or verified as such by recognized certification bodies. To receive halal certification, certain guidelines must be followed during all stages of food production, including slaughtering, storing, processing, marketing, and general hygiene. Worldwide, about 1.6 billion Muslims comprise 23% of the total population (Wilkins et al., 2019). For most Muslims, the halal food protocol is of great importance. Only halal products, which are permitted by Islamic law, are consumed by faithful Muslims (Haque et al., 2015). Muslim customers show more positive views and intents toward halal foods than nonhalal products. The international halal food sector constitutes approximately US$632 billion annually, or 16% of all food consumed globally (Said & Hassan, 2015). Restaurants and fast food manufacturers are the slowest to join halal marketplaces, although major food producers like Nestlé and Unilever have been selling halal items for years. Consumers are more concerned with the authenticity and supply of food as they have become more educated and wealthier. Religion can also impact someone's eating behaviors and responsibilities (Navarro‐Prado et al., 2017).

The halal assurance system (HAS) is a set of vital processes guaranteeing halal compliance from farm to fork. As a result of the development in science and technology, particularly in the food processing business, Muslims now use a variety of components in food products, including gelatin, genetically modified (GM) foods, and medicinal products (Rohman & Windarsih, 2020). They must fulfill the requirements of the halal food supply chain as well as any national standards established by halal organizations like the Central Islamic Council of Thailand (CICOT), Majlis Ulema Indonesia (MUI), Majlis Ugama Islam Singapura (MUIS), and Jabatan Kemajuan Islam Malaysia (JAKIM) (Kwag & Ko, 2019). Numerous efforts are currently being undertaken to generate halal authentication detecting systems that are more efficient (Ng et al., 2022). Over the years, several studies have been conducted to enhance halal identification techniques. Before the advent of polymerase chain reaction (PCR), conventional methods like electrophoresis and dielectric were used to identify halal components. Since its creation, PCR has been widely used for electrophoresis. Additionally, spectroscopic methods have been employed for decades and have recently gained popularity when combined with chemo‐metrics for the processing and handling of data.

The persistence of severe halal adulteration issues on a global scale raises considerable concerns among Muslim customers. The previously mentioned problems involve the inaccurate categorization of nonhalal items as halal, the replacement of halal components with haram substitutes, and inadequate monitoring within the halal supply network. In the context of Pakistan, a nation characterized by a significant Muslim demographic, this matter assumes urgency. The local Muslim community has expressed concern and called for enhanced regulation and enforcement in response to the emergence of several unethical practices. These practices include the utilization of counterfeit halal certificates and the inclusion of nonhalal elements, such as pork and alcohol, in items that are promoted as halal.

The halal assurance system (HAS) is a set of vital processes that guarantee halal compliance from farm to table. To decrease risk during food supply, HAS develops a resilient halal system through effective halal control procedures (Puspaningtyas & Sucipto, 2021). A technique based on hazard analysis and critical control points (HACCP) was suggested to distinguish between likely haram ingredients and halal critical control points.

The HACCP methodology was utilized to determine halal control points at a meat slaughterhouse, while Van der Spiegel et al. (2012) identified halal critical control points in the meat processing industry. Mohamed et al. (2022)) examined the critical control point to ensure halal certification throughout the supply chain. Similar studies have been done on applying HACCP to Islamic dietary law by Qader et al. (2022)), who also identified important elements that influence halal food facilities (Mohd Nawawi et al., 2019). The halal assurance and liability quality system (HAL‐Q) was enacted to achieve halal assurance in food operations (Dahlan et al., 2013). Most importantly, halal food enterprises must understand the points of vulnerability and where preventive and corrective measures should be taken. Still, not many halal control approaches or methods may be applied to detect issues, crucial details, and priority areas of development. This review will look at the latest advances, emerging methodologies, and their applications in halal certification. Also, the accuracy of each method will be assessed to offer valuable insights for further studies.

## 2 FOODS THAT NEED HALAL AUTHENTICATION

Since halal foods are now widely accessible, both Muslims and non‐Muslims are more concerned about the legitimacy of halal food products (Kurniadi & Frediansyah, 2017). All forms of food, especially meat items, must have halal certification. This is because meat products are a significant element necessary for a human diet. Both food and medicines are an inevitable part of human beings. Along with technological progress in biochemistry, globalization, and industrialization, these products must meet consumer demand. Advanced technology has contributed immensely to the field of food science and technology. However, some producers take advantage of this development, and by adding nonhalal ingredients, frauds with the target consumers. Some food products are not labeled correctly regarding the ingredient's source (Montowska & Pospiech, 2010). Even some packaged food is not halal certified. Several food products are questionable and need identification (Table 1).

**TABLE 1:** Questionable ingredients and doubtful sources (Al‐Teinaz, 2020).

Doubtful ingredients	Possible halal/nonhalal sources
Glycerol/glycerin	Saponification of animal fats
Dairy ingredients	Milk source
Taurine	Derived from pig gall
Alcoholic drinks	Any source
Pepsin, clarifiers, and stabilizers	Whether it has been taken from halal/haram source
Emulsifiers	From animals
Enzymes	Microbial, animal, biotechnological
Gelatin	Beef, pork, fish

Meat and products made from meat are very popular around the world because they are an excellent source of several nutrients, including high‐quality proteins, vital amino acids, vitamins, and minerals (Hossain et al., 2021).

Gelatin is commonly used in food products, pharmaceuticals, and cosmetics due to its gelling properties. It also possesses stabilizing, coating, ointment, and healing properties broadly used in commercial consumer goods (Zin et al., 2021).

Alcohol's contribution to the pharmaceutical, food, and cosmetic industries is undeniable; that is why, the halal status of alcohol also needs consideration. The presence of ethanol is a controversial issue (Khattak et al., 2011). Ethanol is justified as Haram by Islamic jurists. However, some beverages contain a certain amount of alcohol that needs to be identified (Alzeer & Abou Hadeed, 2016).

### 2.1 Meat and meat products

With rising demand and costs, meat and meat products seem more vulnerable to unauthorized adulteration, substitution, and false advertising. For consumers who have religious restrictions on the consumption of pork meat, counterfeit meat products are a big problem because of their low cost (He & Yang, 2018). Muslim customers find it annoying when beef meat is combined with or substituted with pig meat; this situation is extremely faced in non‐Muslim countries, making it challenging to get meals certified as halal. Halal foods must be free of all traces of pork and its by‐products. However, eating pork or other edible pig parts is prohibited by Islamic dietary law (Hasimet al., 2022). These foods are strictly forbidden and are referred to as “haram” in Islam.

**FIGURE 1:**
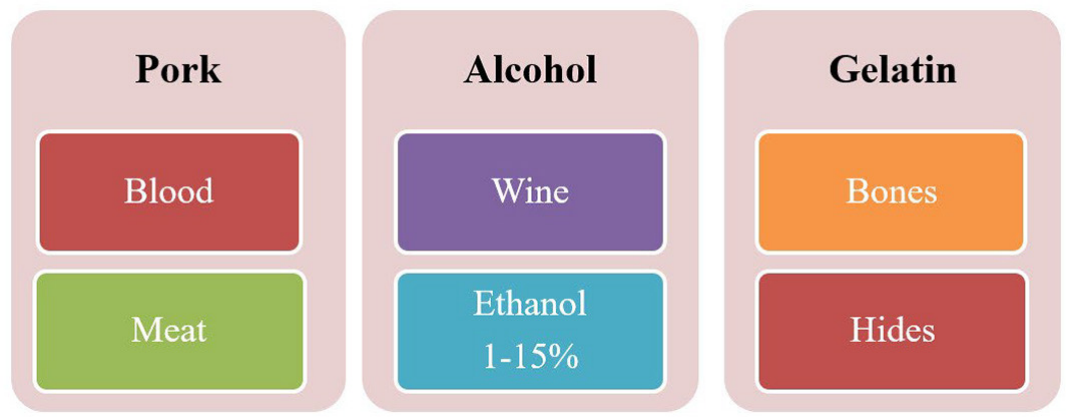
Sources of nonhalal ingredients.

Meat is a major concern regarding halal food; the animal and bird meat that Muslims are permitted to eat is known as halal meat according to Islamic regulations. Various approaches are taken to differentiate pork meat (not halal) from meat products (Kesmen et al., 2007; Rastogi et al., 2007). The price difference is another reason that producers are opting for the unethical adulteration of beef (halal meat) with rat meat (nonhalal meat) (Lestari et al., 2022). Food processing industrialization also exposes Muslims to the ingredients, such as the introduction of nonhalal ingredients (gelatin, blood plasma, and transglutaminase) in the surimi products and meatballs (Sahilah et al., 2016; Figure 1).

The biggest problem in the meat industry is determining the authenticity of food because halal meat products are commonly contaminated with haram food items like meatballs and frankfurters (Table 2). A greater understanding of meat identification would assure product authenticity, safeguard customer safety, and adhere to religious and international norms. The quality and transparency of meat products cannot be guaranteed, even though labeling enables customers to keep track of food specifics and is essential for food safety.

**TABLE 2:** Types of foods that need halal authentication, and concerning methods.

Food types	Need for halal authentication	Analytical authentication method	References
Meats	distinguish pork from all other types of meats	PCR (polymerase chain reaction)	Murugaiah et al. (2009)
Seasonings	Alcohols were produced during fermentation	Mass spectrometry + E‐Nose	Park et al. (2017)
Meatballs and sausages	Pork is used as a substitute	GCMS‐HS + E‐Nose	Nurjuliana et al. (2011)
GM food	Muslims are concerned about genetically modified food	PCR (polymerase chain reaction)	Nikolić et al. (2009)
Confectionery products	Possibility of gelatin taken from pork bones	PCR (polymerase chain reaction)	Sultana et al. (2018)

### 2.2 Beverages or drinks

Alcohol, specifically ethyl alcohol, is frequently used in food production or can be produced due to food processing (Riaz & Chaudry, 2015).

### 2.3 Gelatin

It is a common ingredient in confectionery items like candies, gummies, pastilles, and marshmallows (Uddin et al., 2021). Bovine and porcine are often used types of gelatin. As per scholars' interpretations, porcine gelatin is haram (nonhalal), and certain ailments (bovine spongiform encephalopathy) are associated with bovine gelatin; hence, reliable methods are needed to identify them (Rohman et al., 2020). Additionally, gelatin is employed in pharmaceutical items like capsules because it shields pharmaceuticals from adverse effects like light and oxygen (Zhang et al., 2013).

### 2.4 Genetically modified foods

Halal food consumers are greatly interested in GM foods (Ismail & Mustafar, 2018). Food derived from GM organisms is called GM food and is relatively new food technology. An organism with a genetically modified genome (GM genome) expresses its desired physiological features by altering or replacing its genome (Majid et al., 2015). As per the regulations in Malaysia, it is considered that genetically modified foods do not comply with halal standards. In order to adhere to halal standards, it is necessary for halal food products to be free of genetically modified constituents (Kadafi & Putra, 2021). E‐nose is frequently employed during a food analysis from a halal perspective instead of PCR because sample preparation is more difficult and takes longer with PCR.

## 3 CONVENTIONAL METHODS FOR THE AUTHENTICATION OF HALAL FOODS

### 3.1 Physicochemical method (dielectric)

By monitoring the interaction of food components with electromagnetic radiation, it is possible to differentiate between halal and nonhalal meat and between alcohol and nonalcoholic drinks. Simply, grounded meat and beverages can be measured for their dielectric permittivity by exposing them to a voltage (Abidin et al., 2014). Neither heat nor solvents are required for this method. As proteins become less soluble in liquids when heated, the presence of a solvent could cause a meat protein's three‐dimensional polypeptide chain to break. It has been stated that dielectric characteristics can be used to differentiate various types of meat, notably chicken, pork, and beef, in the range of frequency 0.5 to 50 GHz (Zainal Abidin et al., 2016). Before measuring dielectric, the meats are homogenized and then sterilized using Agilent N5245A PNA‐X. After that, the dielectric constant and dielectric loss factor were obtained by slowly introducing an open‐ended probe to a meat sample within a beaker. To identify pig from chicken and beef, a variation was noticed below 20 GHz, and two separate peaks at 7.43 and 31.19 GHz were observed. Although the studies have not defined any particular target analytes, the two unique peaks may be caused by molecules that are present only in pigs, like DNA, amino acids, proteins, bacteria, enzymes, and others. In addition to meat products, dielectric characteristics have also been studied to detect alcohol content in beverages within the same frequency range as in prior studies (Abidin et al., 2014). This technique is quick, easy, and affordable, with a meat differentiation accuracy of roughly 95% and a LOD of 0.5% alcoholic content in a water mixture. Before measurement, meat items are sterilized at 121°C (Barbosa‐Cánovas et al., 2014). Such a high temperature can destroy harmful pathogens, a crucial factor to consider while performing a halal analysis. However, this method only yields little information. Although different peaks can distinguish pig from chicken and beef, it is uncertain what biomarkers contribute to the peaks.

### 3.2 Electrophoresis

During electrophoresis, charged particles flow through a gel under the influence of an electric field. Meat from different animal species has been distinguished using polysaccharide gel electrophoresis (Ng et al., 2022). Meat extracts were subjected to polysaccharide gel electrophoresis on a glass tube for around 40 min for each sample. After that, the gel was taken off, and bands of myoglobin with natural colors were fixed. The outcomes showed that each meat's protein bands or patterns are distinct and particular. The *R*_B_ value (migration distance for myoglobin bands) for pig species is 0.52 (Ng et al., 2022). DNA fragments are separated based on size using gel electrophoresis and other tools like PCR. Before PCR, different meat species were identified solely by electrophoresis. The meat samples are minced in a mortar and then centrifuged to get a supernatant for electrophoresis. During the application of the PCR, DNA was extracted from meat samples and amplified for electrophoresis (Zhao et al., 2018). Therefore, the proteins present in each meat type, like myoglobin, serve as the basis for the different electrophoretic bands. Another experiment on meat identification (chicken, hog, beef, and turkey) used thin‐layer agarose gel electrophoresis to examine various binary mixes of each meat. The respective ratios of the bands can help determine the quantity of particular meat, as they correlate to the percentage of binary mixtures (Kim & Shelef, 1986; Mafra et al., 2022).

The difference between porcine and bovine gelatin capsules was also determined using SDS‐PAGE gel electrophoresis. The capsules were completely dissolved in water. Then, ammonium sulfate was added to precipitate desired proteins for gelatin source identification selectively. After that, a polyacrylamide gel was loaded with the supernatant. It was electrophoresed for 50 min at 200 V. Depending upon the relative sizes of 140 and 110 kDa, porcine and bovine gelatins were separated from one another. The accuracy of this strategy was said to be 100%. The procedure is easy and cheap because it requires no expensive equipment. However, it requires a lot of time and effort, as it takes 1–2 h to finish. Additionally, this method can only offer a few quantification parameters.

## 4 MODERN METHODS FOR HALAL FOOD AUTHENTICATION

### 4.1 Fourier transform infrared spectroscopy

Fourier transform infrared spectroscopy (FTIR) is a modern method used to detect food contamination from a halal authentication perspective. FTIR technique can acquire an infrared spectrum of absorption, emission, and photoconductivity of solid, liquid, or gas (Candoğan et al., 2021). The FTIR data can be used to identify different functional groups in the sample products. Near‐infrared spectroscopy (14,000–4000 cm^−1^), mid‐infrared spectroscopy (4000–400 cm^−1^), and far‐infrared spectroscopy (400–50 cm^−1^) are some of the FTIR techniques that have been commonly used (Domingos et al., 2012; Esteves et al., 2013).

**FIGURE 2:**
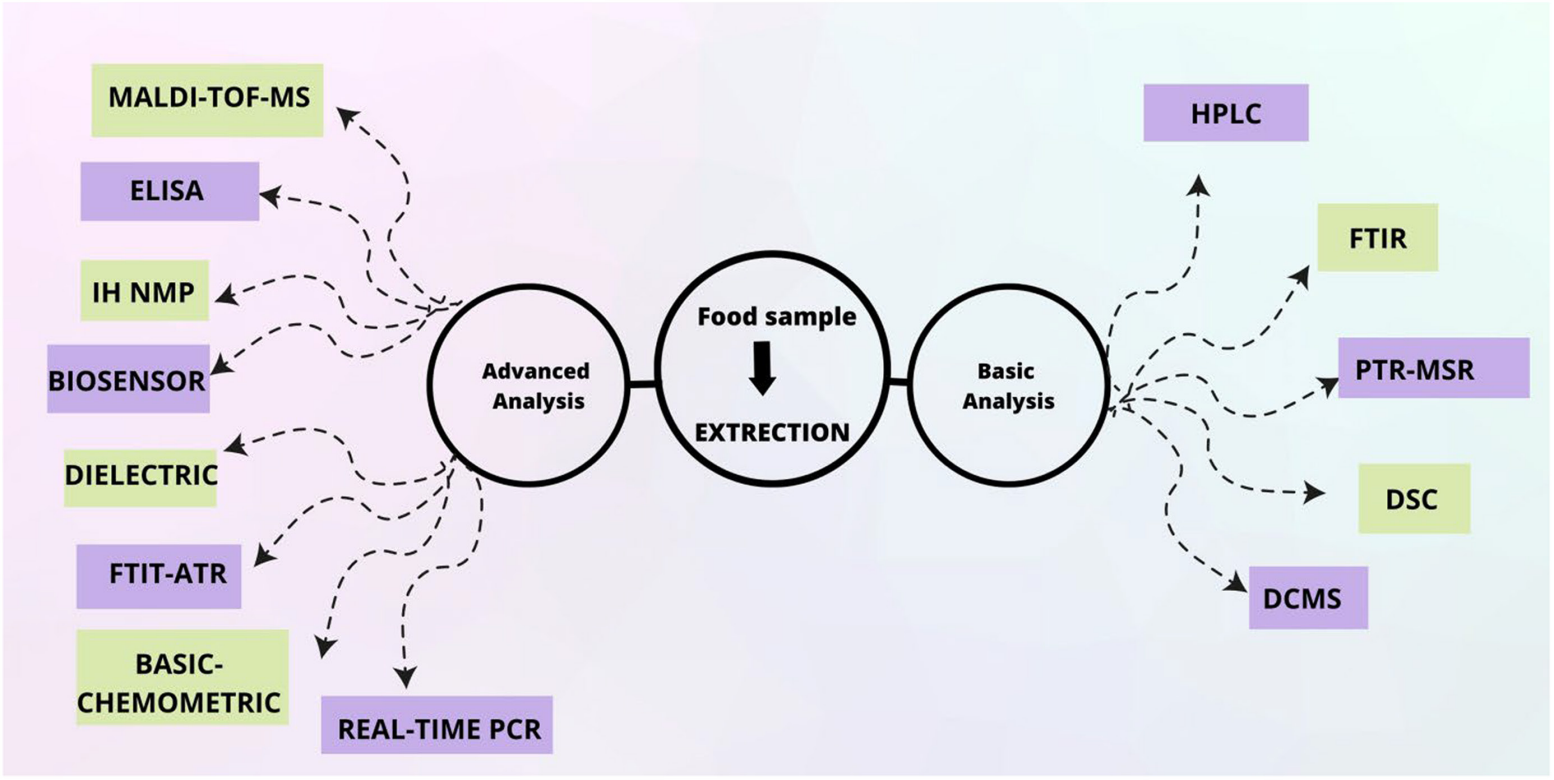
Modern techniques for halal food authentication.

Infrared spectroscopy is an ideal substitute for conventional analytical techniques for nonhalal ingredient inspection. Its minimal sample preparation requirements, restricted use of potentially harmful solvents, high sensitivity, and specificity make it suitable to be used as a fingerprint technique (Domingos et al., 2012). A summary of technically advanced and basic FTIR analysis for halal certification of food and beverage products can be found in Table 3. The modern techniques for halal authentication are shown in Figure 2.

**TABLE 3:** Modern techniques and their required validation parameters.

Foods that can be analyzed	Modern techniques	Subtypes of corresponding techniques	Required validations parameters	References
Ramen stock powder enriched Pork gelatin	PCR	Real‐time PCR	DNA isolation required Selection of primer needed Analysis required (for identification of specific species)	Kang et al. (2018)
Chicken, cow, sheep, and pig meat		PCR‐RFLP	DNA isolation required Primer selection needed Electrophoresis required	Aida et al. (2005)
Fish gelatin, duck, pigeon, rabbit, gummy, candy, and marshmallows		Multiplex PCR	DNA isolation required Primer selection needed PCR assay required	Sultana et al. (2018)
Hot dogs, bacon, Chinese cured pork, and sausage	GC	GC‐CI‐MS	Pretreatment of the sample (in meat samples, sodium nitrate added) (for obtaining supernatant Microwave‐assisted extraction used)	Huang et al. (2013)
Chocolate made of pig adipose tissue		GC–MS	Sample preparation needed (Soxhlet extraction required)	Suparman (2015)
Kimchi, soybean sauce, and gochujang			Sample preparation required (Soxhlet extraction by DMSO)	Nurjuliana et al. (2011)
Mutton, Chicken, and beef		GCMS‐HS	Sample preparation needed Sample pretreatment required (storage at freezing temperature to avoid deterioration) Statistical analysis required	Mortas et al. (2022)
Horse, Pork, beef meat	HPLC	HPLC‐MS/MS	Sample preparation required (mincing and heating of meat) Extraction required (to get supernatant) HPLC analysis required	von Bargen et al. (2014)
Bovine, porcine, and fish (gelatin)		RP‐HPLC‐PCA	Sample preparation required (in the oven by hydrolysis)	Azilawati et al. (2015)
Ham sausages (Halal or Nonhalal)	Molecular spectroscopy	FTIR‐chemometrics	Sample preparation required (fine grinding with KBr) FTIR analysis needed Statistical analysis required	Xu et al. (2012)
Pork, beef		FTIR‐PLS	Sample preparation required (fine grinding of samples) Extraction required (Soxhlet) Analysis required (FTIR and PLS)	Rohman and Windarsih (2020)
Fruit juices and alcoholic beverages	E‐nose	E‐nose‐machine learning	Sample preparation needed (mixing of fruit juices to alcohol in suitable ration) Sample analysis (Cyranose 320) needed for aroma identification	Ordukaya and Karlik (2016)
Soya sauce containing ethanol		E‐nose‐MS	Sample preparation (blending of soy sauce samples) Gas components in the sample need to be analyzed via an e‐nose system	Park et al. (2017)
Meat products		E‐nose‐PCA	Sample pretreatment requires (prevent aroma alteration) precooking in the water bath. Sample analysis and statistical analysis needed	Nurjuliana et al. (2011)
Fermented beverages	Biosensor Technology	PANI film‐AOx	SYNTHESIS of PANI film required Immobilization of AOx enzyme on PANI film needed Detection of ethanol (from green to blue by color variation)	Kuswandi et al. (2014)
Burger meat		AuNPs‐DNA	Synthesis of colloidal AuNPs required Preparation of nano‐bioprobe required (AuNPs and oligoprobe in 1:3), isolation required (DNA), measurement required (via fluorescent)	Ali et al. (2012)
Meat mixtures		AuNP‐swine‐specific nucleotide	Preparation of AuNPs required A colorimeter is needed (for swine‐specific nucleotide detection)	Subara and Jaswir (2018)

### 4.2 Polymerase chain reaction

Polymerase chain reaction (PCR) is a molecular process that amplifies a single or desired number of DNA sequences over multiple orders, producing thousands and millions of copies (Zhu et al., 2020). For ensuring nonhalal ingredients even after food preparation, DNA is often stable and can be found in many food products. In PCR, specially designed primers amplify a specific target sequence (Table 3). In addition, the incorporation of an internal hybridization probe can improve the specificity and sensitivity of PCR (Zhu et al., 2022). This strategy is fast and specific to detect species adulteration with prohibited or nonhalal foods or ingredients. PCR chemistry relies upon the complementary base pairing of the nucleotides within a DNA double helix. When a DNA molecule is heated sufficiently, it denatures or separates into two single strands, as hydrogen bonds holding a double helix together are broken.

### 4.3 Gas chromatography

Other names for gas chromatography (GC) include vapor phase chromatography (VPC) and gas–liquid partition chromatography (GLPC). In utilizing for halal food authentication purposes due to low molecular weight and higher flow rates, detectors, e.g., thermal conductivity detectors (TCD), mass analyzers (MS), electron capture detectors (ECD), and flame ionization detector (FID) result in a reduced analysis time and decreased sample elution temperature (Otles & Ozyurt, 2015; Ribeiro et al., 2020). A wide application of GC in halal food analysis involves qualitative and quantitative analysis, such as composition, food additives, and nonhalal contaminants in various food products. To evaluate halal authentication, gas chromatography may detect many volatile substances, including alcohols, aldehydes, ketones, fatty acids, esters, hydrocarbons, ethers, aromatic, and alicyclic compounds (Cheung & Mehta, 2015; Riu‐Aumatell et al., 2014). Fatty acids are often detected in various oils and fats using gas chromatography and flame ionization detection (GC‐FID). Following standard protocols, it may detect adulteration by evaluating the retention period and peak areas of fatty acid methyl esters derived from fatty acids. To check food products from a halal perspective, the composition and structure of fatty acids in oils and fats can be utilized as a marker to determine the origin of lipids (Kamarulzaman et al., 2016; Uddin et al., 2021).

### 4.4 High‐performance liquid chromatography (HPLC)

Modern techniques like HPLC are mostly used for the preparative and analytical separation of different substances found in less or more complex combinations. Due to the multiple advantages of this technique, it has attracted great attention for detecting prohibited contamination or nonhalal adulteration in foods for halal authentication (Verma & Pant, 2021). The most important benefit is that HPLC can easily separate the less volatile sample components. Additionally, it can be applied to detect the origin of components in food systems that are thermally unstable, extremely polar, and have high molecular mass. It also has the advantage that it does not require the derivatization of the analyte.

### 4.5 Differential scanning calorimetry

Differential scanning calorimetry (DSC) is a thermo‐analytical method used to analyze the physical behavior of fats, oils, proteins, carbohydrates, alcohol, water content, and food packaging during processing and storage (Karim & Muhamad, 2018). This technique is utilized to expose butter adulterated with lard, as lard and butter have similar characteristics making lard a desirable adulterant in the butter. Many scientists used this technique to address food adulteration problems and halal authentication.

### 4.6 Proton transfer reaction mass spectroscopy (PTR‐MS)

Low concentrations of volatile organic compounds (VOCs) in gases are measured and monitored in absolute time using an advanced technique called proton transfer reaction mass spectrometry (PTR‐MS) (Rohman et al., 2021). PTR‐MS may be used to quantify the volatile components of animal fats (cow, pig, and milk fat) and vegetable oils (palm kernel, palm, and coconut oil). PTR‐MS provided 89% accurate classifications. It allows measuring at a very high speed compared to other approaches but needs further research. In addition, it concentrates on identifying three by‐products of the fat industry (fish oil, animal fat, and regenerated edible oils) that can be used in animal feeds. PTR‐MS is a reliable technique for approving nonhalal adulteration practices by identifying components in fish oils. The fatty acid fingerprints, triacylglycerol fingerprints, and volatile chemical compound profiles were used to predict their identities related to halal authentication. The benefits and challenges related to modern techniques for halal authentication are presented in Table 4.

**TABLE 4:** Benefits and challenges of modern techniques for halal authentication.

Nonhalal ingredients	Techniques	Advantages	Challenges	Sources
DNA Swine protein	Polymerase chain reaction	Specific and sensitive because DNA is present in all organisms and cells	Specific primers for PCR amplification and high efficiency needed	Kang et al. (2018) Abd Mutalib et al. (2015) Sudjadi et al. (2016)
Volatile compounds (diacetyl, methyl‐propanal, propanal, heptanal, nonanal, trimethyl pyrazine)	Electronic nose	Low cost As compared to GCMS, HPLC, and IR Accurate and fast detection Eco‐friendly Not complex It can be coupled with other techniques	The aroma can be changed samples pretreatment not done to avoid result changes. Proper storage needed (−20°C)	Sarno et al. (2020) Wang et al. (2019) Nurjuliana et al. (2011)
In meat (N‐nitrosamines, fatty acids contents	Gas chromatography	It gives highly accurate results Separating gases from samples Complex systems can provide analysis of a broad range of components	During sample preparation, loss of volatile compounds For analysis suitable column selection needed	Sarno et al. (2020) Lehotay and Hajšlová (2002) Cajka (2013)
Ethanol in liquid drinks DNA of Pork	Biosensors	Low cost Highly sensitive Less time required Electrophoresis is not required as in conventional PCR Suitable for on‐site monitoring	The stability of biosensors has been questioned in real samples Results inconsistency	Ali et al. (2012) Zia et al. (2020) Kuswandi et al. (2014)
Porcine amino acids	High‐performance liquid chromatography	Speedy detection takes approximately 30 min Simple analysis Specific for targeted analytes	For derivatization need a suitable fluorophore or chromophore	Azilawati et al. (2015) von Bargen et al. (2014) Azilawati et al. (2015)
Not specified	Bacterial count	Expensive instruments are not required as it uses CPC or SPC plates for counting bacterial colonies. Simple procedure Low cost	To compare CPC and SPC, halal and nonhalal samples should be in the same environment.	Sabow et al. (2015) Hakim et al.(2020)

### 4.7 Biosensor

#### 4.7.1 Electronic nose

An electronic nose (E‐nose) is a tool or detecting device which resembles the human body's natural olfactory sense. It is developed based on the interaction of semiselective sensors with volatile substances, which results in a physical change inside the sensor as volatile substances adsorb to its surface (Di Rosa et al., 2017). It was utilized in the food sector for freshness and maturity testing, quality control of raw and processed commodities, shelf‐life analysis, and authenticity tests (particularly for halal food confirmation). To find and measure meat adulterants, E‐nose was employed in conjunction with a gas chromatography–mass spectrometer (GC–MS) (Wang et al., 2019). The GC–MS data's qualitative and quantitative features are connected to the examined E‐nose signals. Hence, the E‐nose is effective for detecting meat adulterants related to the halal issue, and when it is coupled with an MS, it considerably increases this capability. Meat items, culinary oils, spices, and drinks may all be examined with an e‐nose (Di Rosa & Leone, 2018). E‐nose is frequently employed during a food analysis from a halal perspective instead of PCR because sample preparation is more difficult and takes longer with PCR.

According to Nurjuliana et al. (2011), principal component analysis (PCA) and the E‐nose were used to find and analyze the evidence of pork in different meat products. This study's E‐nose was built using a surface acoustic sensor (SAW). The frequency of SAW changes when volatiles penetrate the sensor's surface, which impacts the detection signals. As a result, chemicals may be found and identified using the E‐nose (Nurjuliana et al., 2011). Raw pork has been shown to contain several substances or scents, including the buttery‐smelling diacetyl and 3‐hydroxy‐2‐butanone, the pungent 2‐methyl‐propanal, the fatty‐smelling heptanal, the roasted‐smelling trimethyl pyrazine, and the nonanal and decanal. The Kovats index database can be used to locate the peaks. This approach offers a quick, precise, affordable, and eco‐friendly analysis with a 5‐g sample and an analysis time of less than a minute. To authenticate and verify halal goods, this approach is beneficial for identifying components that include porcine.

#### 4.7.2 Nano‐bioprobe

Functional nanoparticles having covalent or noncovalent bonding with biomolecules, including polynucleotides, proteins, and peptides, make up hybrid biomaterials, which also share dimensions with biopolymers and have size‐dependent optoelectronic capabilities (Ali et al., 2012). Therefore, it is a particularly interesting and promising method for halal food analysis. These conjugated biomaterials include multiple bioassays, structural scaffolds for tissue engineering, long‐term and controlled drug release, ultrasensitive optical sensing and imaging, material synthesis, and in‐vivo magnetic resonance imaging (MRI) (Subara & Jaswir, 2018). A new class of species‐specific nano‐bioprobes was developed to evaluate the absence of pork in ready‐to‐consume food products. They created AluI‐cut segments of the mitochondrial (mt) specific gene by structurally and functionally integrating 27 nucleotides into 3‐nm‐diameter gold nanocrystals coated with citrate tannate. This technique is more affordable than real‐time PCR. It is appropriate for studying heterogeneous samples where the PCR approach is ineffective due to the degradation of longer DNA templates into smaller pieces.

#### 4.7.3 Optical biosensors

Optical biosensors are employed as a helpful technique for halal verification, utilizing alterations in light characteristics to identify specific substances present in food. Biorecognition elements, such as antibodies or enzymes, are responsible for the functioning of these systems (Flauzino et al., 2022). These elements interact with the target substance, resulting in the generation of a quantifiable optical signal. The signal is transformed into a numerical assessment of the concentration of the substance being analyzed. Optical biosensors exhibit notable attributes such as heightened sensitivity, quick response rates, and the ability to be customized for the detection of diverse chemicals pertinent to halal verification, including prohibited additives or impurities. Optical biosensors are widely utilized in the food sector, particularly in nations that enforce rigorous halal certification criteria. These biosensors are employed with the purpose of validating the genuineness of items that bear the halal label, thereby guaranteeing adherence to Islamic dietary regulations (Singh et al., 2023).

#### 4.7.4 Surface plasmon resonance (SPR)

Surface plasmon resonance technology is a robust technique utilized in the certification of halal food. The operational mechanism of this system is based on the detection of alterations in refractive index occurring on a sensor surface because of the interaction between polarized light and a thin metal film, commonly composed of gold (Revathi & Rajeswari, 2022). In case of halal verification, the employment of SPR can be employed to discern and identify chemicals that hold significant importance in adhering to Islamic dietary regulations. These compounds comprise forbidden additives or impurities. The immobilization of biorecognition elements, such as antibodies or aptamers, onto the sensor surface is the method employed to do this. The detection of analytes is facilitated by the quantifiable alterations in SPR signals that occur upon the binding of target molecules. This label‐free and quick detection technology is widely utilized in the field of meat product verification, particularly for the precise identification of nonhalal constituents such as pig. Moreover, this technique is utilized in the examination of processed food and drinks to verify their compliance with halal regulations, becoming a vital component in the halal food sector (Ng et al., 2022).

### 4.8 Nuclear magnetic resonance (NMR) spectroscopy

Nuclear magnetic resonance (NMR) spectroscopy is a robust method, which can rapidly analyze mixtures at the molecular level without requiring separation and/or purification steps, making it ideal for applications in food analysis.

Nuclear magnetic resonance is used by spectroscopic methods such as proton nuclear magnetic resonance (hydrogen1 NMR or proton NMR) to identify the structural characteristics of a material. NMR can quickly analyze the mixture at the molecular level without separating or purification steps, making it suitable for food analysis (Hatzakis, 2019). In comparison with other chromatographic methods coupled with mass spectrometry, NMR spectroscopy has widely been used in the certification of olive oil due to its reproducibility and nondestructive nature (Fang et al., 2013). To examine the halal authenticity of 10 different vegetable oils, including olive oil, extra virgin olive oil, soybean, canola, corn, palm, sunflower, coconut, rice bran, and peanut oil, Fang et al. (2013)) used GC/MS fingerprinting, 1H NMR spectroscopy, and chemometrics with heating fats (such as adipose tissues of chicken, beef, mutton, and pig). However, the NMR study did not establish a successful and good characterization in the context of specificity and sensitivity, particularly in comparison with GC/MS data applying orthogonal projections to latent structures discriminant analysis (OPLS‐DA) and partial least squares discriminant analysis (PLS‐DA) as prediction models. Additionally, it has been suggested that PLS models may successfully detect vegetable oil that has been spiked with as little as 5% fat or beef tallow (Fang et al., 2013).

### 4.9 Competitive indirect ELISA

Enzyme‐linked immunosorbent assay (ELISA) is a typical immunological technique for determining antigens, antibodies, and protein levels in various biological specimens (Kim & Shelef, 1986). Tukiran et al. (2016) designed a competitive indirect ELISA for the quick identification of porcine gelatin present in the edible bird's nest (EBN) linked with a halal authentication perspective. Polyclonal rabbit antibodies were employed to create three ELISAs against collagen a1 (I) chain (pAb3) and collagen a2 (I) chain (pAb1 and pAb2) amino acid sequences specific to porcine species. The detection limits (IC15) for these ELISAs are 0.052, 0.033, and 0.082 (mg/mL), respectively. Bovine and porcine gelatins are also recognized by pAb1, pAb2, and pAb3, which had median inhibitory concentrations (IC50) of 0.265, 0.394, and 0.228 (mg/mL), respectively. Cave nest and egg white were slightly cross‐reactive to pAb1, and blood cave nest and egg white were slightly cross‐reactive to pAb2. Moreover, no cross‐reaction was observed between the pAb3 and EBNs or egg white. While using pAb3, recovery of EBNs spiked with porcine gelatin ranges from 62.8 to 125.4% with intra‐ and interday coefficients of variation (CVs) of 2.9 to 5.4 and 4.7 to 9.6, respectively. The pAb3 is, therefore, suggested as being adequate for EBN authentication.

### 4.10 Dielectric properties from microwave

For the noninvasive characterization of food and its constituents, a desirable and more effective strategy is to use electromagnetic radiation found in the microwave. The dielectric characteristics of a material, which are made up of the dielectric constant and dielectric loss, are information obtained from the interaction of various materials with microwave electromagnetic energy. Numerous studies have published an extensive summary of the dielectric characteristics of various materials at microwave frequencies (Gezahegn et al., 2021). Dielectric characteristics have been used to describe the food water content, sugar content, acid content, and quality of grape juice and wine, among other aspects of food quality. Compared to current lab‐based techniques like FTIR and PCR, this dielectric measurement and approach offer a straightforward, efficient, reliable, and nonlaborious alternative. Utilizing this dielectric measurement might speed up decision‐making processes and considerably decrease the time needed to analyze an item to determine if it is halal (Abidin et al., 2014; Ali et al., 2021). A viable approach for identifying and distinguishing alcoholic beverages for halal verification has been proposed by Abidin et al. (2014)) using dielectric characteristics. For verification, the behavior of several alcoholic solutions was examined across a microwave range of 0.5–50 GHz. Multiple commercially available alcoholic drinks were included in the measurements. From their research, they were able to demonstrate that, at frequencies between 10 and 25 GHz, the alcohol content may be distinguished by dielectric characteristics down to the minimum observed 0.5% concentration in a water combination. Beyond this threshold, solutions are regarded as alcoholic beverages.

### 4.11 Matrix‐assisted laser desorption ionization–time‐of‐flight mass spectrometry

Matrix‐assisted laser desorption ionization–time‐of‐flight mass spectrometry (MALDI–TOF MS), an emerging technology, is used for protein profiling and species identification. This technique is more innovative, reliable, inexpensive, simpler, and faster than other phenotypic and molecular techniques used to identify human pathogenic microbes. Flaudrops et al. (2015)) examined the nature of gelatin and meat (chicken, hog, horse, beef, and veal) using MALDI–TOF MS techniques. It can detect up to 1% gelatin in candy that has been spiked, and up to 20% pig gelatin can be found in beef gelatin. This approach is important for meat and collagen's routine and comprehensive detectability.

### 4.12 Isotope analysis

Isotope analysis is a reliable method utilized for the purpose of halal authentication, namely, in the process of confirming the source and content of food items (Kua et al., 2022). The approach employed in this methodology is predicated upon the assessment of stable isotopes, which are distinct forms of elements characterized by the same number of protons but varying neutron quantities. The geographical or biological origin of a food item can be traced by conducting an analysis of the isotopic composition of important elements, such as carbon, nitrogen, and oxygen. The isotopic composition of water and minerals assimilated by plants exhibits region‐specific characteristics. The isotopic composition of animal tissues can provide insights into their dietary habits, including the distinction between consumption of halal or nonhalal feed. This methodology is widely utilized for the purpose of verifying the legitimacy of halal meat products, particularly in areas where there are strict regulations for halal certification (Zhao et al., 2020).

## 5 CONCLUSION AND FUTURE PERSPECTIVES

Various advanced analysis methods have recently been evaluated for their conformity with food certification, addressing halal foods affected by food adulteration. Skilled human experts have formerly carried out examinations of halal food authentication. However, these traditional techniques have multiple risks, prolonged procedures, high costs, and are far more subjective. Recent advanced approaches provide various advantages over traditional techniques, with speed, accuracy, reliability, objectivity, eco‐friendliness, and absence of pattern pretreatment. This review concludes that biosensors will be the largest point‐of‐care device for precise and quick detection of foods containing alcohol, pork, and other banned meats (like donkeys and horses) and any slaughtered animals that do not follow the halal slaughter method. Compared to other advanced technologies, biosensors are inexpensive and quick to detect. They can also be made smaller, so that absolute and on‐site detection is possible, allowing them to be used in major international events. However, there are certain limitations as the achieving of a considerable degree of specificity and sensitivity in biosensors for the detection of minute quantities of banned chemicals, particularly within diverse food matrices, is a notable obstacle that may result in the occurrence of false positives or unclear outcomes. Moreover, the incorporation of biosensors for halal food authentication is further complicated by the potential risk of cross‐reactivity, which refers to the possibility of biosensors detecting substances other than the intended analytes. This challenge is further complicated by the need for standardized protocols, certification processes, and the consideration of ethical and cultural factors. The storage stability of biosensors for precise sample analysis needs to be further improved to guarantee the accuracy and authenticity of the analysis for certifying halal foods. Prospects must consider the inclusion of AI in the assessment of halal food certification to increase speed, accuracy, and the capacity to detect multiplexed analytes.

## AUTHOR CONTRIBUTIONS

**Ifrah Usman:** Writing – original draft (equal); writing – review and editing (equal). **Saima Sana:** Formal analysis (equal); writing – review and editing (equal). **Muhammad Afzaal:** Supervision (equal); writing – original draft (equal). **Ali Imran:** Formal analysis (equal); validation (equal). **Farhan Saeed:** Project administration (equal); validation (equal). **Aftab Ahmed:** Formal analysis (equal); validation (equal). **Yasir Abbas Shah:** Writing – original draft (equal); writing – review and editing (equal). **Muniba Munir:** Data curation (equal); writing – review and editing (equal). **Huda Ateeq:** Formal analysis (equal); validation (equal). **Atka Afzal:** Data curation (equal); investigation (equal). **Iqra Azam:** Data curation (equal); investigation (equal). **Afaf Ejaz:** Data curation (equal); writing – review and editing (equal). **Gulzar Ahmad Nayik:** Resources (equal); writing – review and editing (equal). **Mahbubur Rahman Khan:** Formal analysis (equal); writing – review and editing (equal).

## CONFLICT OF INTEREST STATEMENT

The authors declare no conflict of interest.

## ETHICS STATEMENT

The study involved no experimentation with human subjects.

## CONSENT TO PARTICIPATE

The authors declare their consent to participate in this article.

## CONSENT TO PUBLISH

The authors declare their consent to publish this article.

## Data Availability

Even though adequate data have been given in the form of tables and figures, all authors declare that if more data are required, then the data will be provided on a request basis.
